# Association of Alcohol Screening Scores With Adverse Mental Health Conditions and Substance Use Among US Adults

**DOI:** 10.1001/jamanetworkopen.2020.0895

**Published:** 2020-03-12

**Authors:** Maria R. Khan, Kailyn E. Young, Ellen C. Caniglia, David A. Fiellin, Stephen A. Maisto, Brandon D. L. Marshall, E. Jennifer Edelman, Julie R. Gaither, Natalie E. Chichetto, Janet Tate, Kendall J. Bryant, MacRegga Severe, Elizabeth R. Stevens, Amy Justice, Scott R. Braithwaite

**Affiliations:** 1 Department of Population Health, New York University School of Medicine, New York; 2 Department of Internal Medicine, Yale School of Medicine, New Haven, Connecticut; 3 Department of Psychology, Syracuse University, Syracuse, New York; 4 Brown University School of Public Health, Providence, Rhode Island; 5 Division of General Internal Medicine and Public Health, Vanderbilt University Medical Center, Nashville, Tennessee; 6 National Institutes of Health, Bethesda, Maryland

## Abstract

**Importance:**

Alcohol screening may be associated with health outcomes that cluster with alcohol use (ie, alcohol-clustering conditions), including depression, anxiety, and use of tobacco, marijuana, and illicit drugs.

**Objective:**

To quantify the extent to which alcohol screening provides additional information regarding alcohol-clustering conditions and to compare 2 alcohol use screening tools commonly used for this purpose.

**Design, Setting, and Participants:**

This longitudinal cohort study used data from the Veterans Aging Cohort Study. Data were collected at 8 Veterans Health Administration facilities from 2003 through 2012. A total of 7510 participants were enrolled, completed a baseline survey, and were followed up. Veterans with HIV were matched with controls without HIV by age, race, sex, and site of care. Data were analyzed from January 2019 to December 2019.

**Exposures:**

The Alcohol Use Disorders Identification Test (AUDIT) and Alcohol Use Disorders Identification Test–Consumption (AUDIT-C) were used to assess alcohol use, with 4 risk groups delineated for each test: score 0 to 7 (reference), score 8 to 15, score 16 to 19, and score 20 to 40 (maximum score) for the full AUDIT and score 0 to 3 (reference), score 4 to 5, score 6 to 7, and score 8 to 12 (maximum score) for the AUDIT-C.

**Main Outcomes and Measures:**

Alcohol-clustering conditions, including self-reported symptoms of depression and anxiety and use of tobacco, marijuana, cocaine, other stimulants, opioids, and injection drugs.

**Results:**

A total of 6431 US patients (6104 [95%] men; median age during survey years 2003-2004, 50 years [range, 28-86 years; interquartile range, 44-55 years]) receiving care in the Veterans Health Administration completed 1 or more follow-up surveys when the AUDIT was administered and were included in the present analyses. Of the male participants, 4271 (66%) were African American, 1498 (24%) were white, and 590 (9%) were Hispanic. The AUDIT and AUDIT-C scores were associated with each alcohol-clustering condition. In particular, an AUDIT score of 20 or higher (vs <8, the reference) was associated with symptoms of depression (odds ratio [OR], 8.37; 95% CI, 6.20-11.29) and anxiety (OR, 8.98; 95% CI, 6.39-12.60) and with self-reported use of tobacco (OR, 14.64; 95% CI, 8.94-23.98), marijuana (OR, 12.41; 95% CI, 8.61-17.90), crack or cocaine (OR, 39.47; 95% CI, 27.38-56.90), other stimulants (OR, 21.31; 95% CI, 12.73-35.67), and injection drugs (OR, 8.67; 95% CI, 5.32-14.13). An AUDIT score of 20 or higher yielded likelihood ratio (sensitivity / 1 − specificity) values greater than 3.5 for depression, anxiety, crack or cocaine use, and other stimulant use. Associations between AUDIT-C scores and alcohol-clustering conditions were more modest.

**Conclusions and Relevance:**

Alcohol screening can inform decisions about further screening and diagnostic assessment for alcohol-clustering conditions, particularly for depression, anxiety, crack or cocaine use, and other stimulant use. Future studies using clinical diagnoses rather than screening tools to assess alcohol-clustering conditions may be warranted.

## Key Points

**Question:**

Can alcohol use screening scores provide clinically meaningful information and facilitate identification of adverse mental health conditions and other substance use?

**Findings:**

This cohort study using data from 6431 US patients collected from 2003 to 2012 found that high alcohol use scores (Alcohol Use Disorders Identification Test score ≥20) were associated with depression, anxiety, crack or cocaine use, and other stimulant use, with likelihood ratios greater than 3.5.

**Meaning:**

These findings suggest that alcohol screening can inform decisions about further screening and diagnostic assessment for depression, anxiety, and some drug use outcomes.

## Teaser

This cohort study examines whether alcohol screening provides information regarding alcohol-clustering conditions, including depression, anxiety, and use of tobacco, marijuana, and illicit drugs among US adults.

## Introduction

The health consequences of alcohol use are substantial, with rates of alcohol use having increased dramatically in the most recent reporting periods.^1,2^ Unhealthy alcohol use encompasses a range of alcohol use patterns, from risky use, which is defined as exceeding the recommended daily drinking limits (ie, >3 drinks per day for women and >4 drinks per day for men),^3^ to harmful use, which is accompanied by alcohol-related consequences (eg, failure to fulfill obligations or interpersonal problems), and dependence, which is accompanied by substantial impairment (eg, tolerance, withdrawal, or inability to reduce alcohol consumption).^4^ In the US, the most recent estimates suggest that 13% of adults exceed recommended daily drinking limits on a weekly basis, representing a 30% increase over a 10-year period, and an additional 13% have alcohol use disorder,^1^ representing a 50% increase over the course of 10 years.^1^ Globally, alcohol use disorder is the most common substance use disorder, with estimates indicating a worldwide population of 100 million individuals with alcohol use disorder.^2^ Current guidelines suggest annual screening and treatment or referral for unhealthy alcohol use in adult primary care settings in an effort to reduce alcohol use–related morbidity and mortality.^5,6^ In many health care systems, alcohol screening is integrated into routine primary care.^7,8^

Depression, anxiety, and the use of substances other than alcohol are highly prevalent in the US.^9,10,11^ The US Preventive Services Task Force recommends screening for depression and tobacco use but does not currently recommend illicit drug use screening (although recent guidelines have been drafted) and does not recommend screening for anxiety.^12^ Screening for recommended conditions may not be feasible for some practitioners because of resource constraints.^13,14^ Given that unhealthy alcohol use is associated with mood and anxiety disorders,^15,16^ as well as use of other substances including tobacco,^17,18^ marijuana,^17^ prescription opioid misuse,^19^ heroin,^20^ crack or cocaine, and other stimulants,^21,22,23,24^ information obtained from alcohol screening may provide clinically meaningful information regarding some or all of these conditions, potentially facilitating their identification and treatment.

Even though alcohol screening has been widely integrated into primary care and could provide insight into risk for other conditions in these settings, research on the association between alcohol use screening scores and other conditions is limited.^25,26^ Although the 10-item Alcohol Use Disorders Identification Test (AUDIT)^27^ is commonly used for alcohol use screening, the Alcohol Use Disorders Identification Test–Consumption (AUDIT-C), an abbreviated 3-item version of the full AUDIT, also reliably identifies unhealthy alcohol use and alcohol use disorder.^28,29,30^ Given the time constraints on primary care physicians,^31^ brief screeners such as the AUDIT-C are useful and practical tools for unhealthy alcohol use screening. However, the more-comprehensive AUDIT may more accurately screen individuals at high risk of alcohol-clustering conditions compared with the AUDIT-C.

The purpose of this study was to evaluate to what extent currently used alcohol screening measures provide information regarding the presence of conditions that are likely alcohol-clustering according to the literature (henceforth referred to as “alcohol-clustering conditions”). Specifically, we used data from the Veterans Aging Cohort Study (VACS), a cohort of veterans with HIV matched to HIV-negative controls, and used the AUDIT and AUDIT-C to measure associations between alcohol use severity and conditions such as depression and anxiety, as well as substance use, including tobacco, marijuana, illicit opioids, stimulants, and injection drugs.

## Methods

### Sample and Data Sources

The VACS survey sample includes US veterans receiving health care in 8 Veterans Health Administration centers: Atlanta, Georgia; Baltimore, Maryland: Bronx, New York; Houston, Texas; Los Angeles, California; Manhattan and Brooklyn, New York; Pittsburgh, Pennsylvania; and Washington, DC. The VACS is composed of approximately 3500 veterans with HIV and 3500 controls without HIV, frequency-matched by age, race/ethnicity, sex, and site of care.^32^ Patients of the Veterans Health Administration self-report their race/ethnicity; these data were used by the VACS study team during matching to ensure comparability of the HIV-positive and HIV-negative cohorts. Enrollment in VACS began in 2002 and is ongoing. The VACS participants provide written informed consent for participation in baseline and follow-up surveys that assess information about a range of health outcomes and health-related sociodemographic and behavioral factors. Participant survey data are matched to clinical and administrative data. Institutional review boards at each participating Veterans Health Administration medical center and affiliated academic institutions approved all parent study activities. The institutional review board of the New York University School of Medicine approved all study activities for the present secondary data analysis study focused on alcohol use screening for the identification of comorbid conditions. We used data from 6 annual surveys that administered the full AUDIT and the AUDIT-C. These surveys were administered from 2003 to 2012 in Atlanta, Bronx, Houston, Los Angeles, Manhattan and Brooklyn, and Pittsburgh and from 2004 to 2012 in Baltimore and Washington, DC. The present study describes results of analysis performed from January 2019 to December 2019. This study follows the Strengthening the Reporting of Observational Studies in Epidemiology (STROBE) reporting guideline.

### Measures

The current analysis considers patterns of alcohol use, defined by specific ranges of scores on the AUDIT and AUDIT-C, as well as symptoms of anxiety and depression and the use of substances other than alcohol. All instruments and thresholds used in our analyses are described in the following subsections.

#### Alcohol Use Patterns Measured by the AUDIT Questionnaire

The AUDIT is a 10-item questionnaire that was designed to detect hazardous or harmful drinking across settings and subgroups.^33,34,35^ The AUDIT assesses 3 domains of alcohol use: past-year consumption based on frequency, quantity, and heavy drinking; past-year dependence symptoms, including impaired control, increased salience of drinking, and morning drinking; and consequences of use (eg, guilt, blackouts, alcohol-related injury, and others’ concern about one’s use). Each item is scored from 0 to 4 for a maximum score of 40. Those reporting no alcohol use in the past year are given a score of 0 on all items with the exception of items 9 and 10, which are not restricted to the past year. On the basis of World Health Organization guidelines,^27^ we categorized AUDIT scores into 4 risk groups (scores 0-7 [reference], 8-15, 16-19, and 20-40). Participants were categorized in the lowest category if they reported never drinking or not drinking in the past year even if missing AUDIT items 9 and/or 10, or if they were missing 1 AUDIT item but the sum of the remaining 9 AUDIT items was less than or equal to 3. Participants were categorized in the highest category if they were missing 1 or more AUDIT items but the remaining items when summed yielded a score of 20 to 40.

#### Alcohol Use Patterns Measured by the AUDIT-C Questionnaire

The AUDIT-C, the first 3 items of the full AUDIT, measures past year consumption patterns according to frequency, quantity, and heavy drinking. The score ranges from 0 to 12. We categorized AUDIT-C scores into 4 risk groups (scores 0-3 [reference], 4-5, 6-7, and 8-12). Our prior studies of the VACS sample,^36^ which used group-based mixture modeling methods to categorize the sample on alcohol use, suggest that 4 distinct alcohol use patterns (abstainer, low risk, moderate risk, and high risk) characterize the sample and that increasing alcohol risk is associated with a dose-response increase in mortality risk. A score of 4 or higher is the standard AUDIT-C cut point indicative of unhealthy alcohol use,^37^ whereas a score of approximately 8 is associated with exposure to biologically confirmed alcohol use, a mortality risk indicator, and trauma-related hospitalizations.^36,38^

#### Alcohol-Clustering Conditions: Psychiatric Disorder Symptoms

Depressive symptoms were measured using the Patient Health Questionnaire–9 (PHQ-9), a 9-item screening instrument that assesses the frequency of experiencing depression-related problems (eg, “little interest or pleasure in doing things” or “feeling down”) with response options rated on a 4-point scale ranging from 0 (“not at all) to 3 (nearly every day”).^39^ In accordance with Kroenke et al,^40^ we used a PHQ-9 score of 10 or more to identify cases of current depressive symptoms.^41^ Anxiety symptoms were assessed by a single survey item that asked whether the participant had “felt nervous or anxious” in the 4 weeks before the survey and, if they had this symptom, the degree to which they were bothered on a 4-point Likert scale.^42,43^ Single-item screening tools for anxiety have shown robust test performance in detection of validated measures of anxiety symptoms.^44^ We coded a dichotomous variable indicating any endorsement of the symptom.

#### Alcohol-Clustering Conditions: Other Substance Use

We examined dichotomous indicators (yes vs no) of current substantial tobacco use (≥10 cigarettes per day) and past-year use of marijuana, crack or cocaine, other stimulants (eg, amphetamine), and illicit opioids, including heroin and/or prescription opioids (eg, Oxycontin, Vicodin, or Percocet; prescription opioids were not assessed during the 2005-2007 survey wave).

#### Screening Using the AUDIT vs Commonly Used Evidence-Based Screening Tools

We compared the test performance of the AUDIT with the following evidence-based tools used commonly in clinical practice: the Patient Health Questionnaire–2 (PHQ-2; first 2 items of the PHQ-9) for indication of depression,^41^ the Generalized Anxiety Disorder 7-item (GAD-7) scale for indication of anxiety,^45,46^ and the Drug Abuse Screen Test–10 (DAST-10) for indication of crack or cocaine use.^47,48^

### Statistical Analysis

All analyses were conducted using Stata statistical software version 15.0 (StataCorp). Bivariable analyses were conducted to describe across-time levels of alcohol use patterns and alcohol-clustering conditions. Using the 6 VACS survey waves (survey wave 2003-2004 to wave 2011-2012), we estimated cross-sectional logistic regression models to estimate unadjusted odds ratios (ORs) and 95% CIs for associations between categories of alcohol use severity and alcohol-clustering conditions, using random effects to account for within-individual clustering across follow-up periods.^49,50,51,52^ We included an alcohol use pattern by HIV status interaction term in each model to test for statistically significant differences in the association between AUDIT or AUDIT-C category and alcohol-clustering condition by HIV status. We assessed the test performance of the AUDIT or AUDIT-C as screening tools for association with alcohol-clustering conditions and evaluated these tools when using different thresholds to define a positive test. Specifically, we calculated the sensitivity, specificity, likelihood ratio (sensitivity / 1 − specificity), positive predictive value (PPV), and the percentage of individuals correctly classified when using alcohol screening for indication of depression, anxiety, and other substance use. Finally, we compared the likelihood ratios obtained when using the AUDIT for indication of depressive symptoms, anxiety symptoms, and crack or cocaine use with likelihood ratios obtained from the PHQ-2 for depressive symptoms,^41^ GAD-7 for anxiety symptoms,^46^ and DAST-10 for crack or cocaine use.^47^ All models used complete case analysis.

## Results

A total of 7510 participants were enrolled, completed a baseline survey, and were followed up. The median age in survey years 2003 to 2004 was 50 years (range, 28-86 years; interquartile range, 44-55 years). Of the participants, 6104 (95%) were men, and 327 (5%) were women. Of the male participants, 4271 (66%) were black, 1498 (24%) were white, 590 (9%) were Hispanic, and 2747 (45%) had an annual income of less than $12 000. The AUDIT was not administered at baseline. A total of 6431 participants (86%) completed 1 or more follow-up surveys, for a total of 22 473 surveys across follow-up, when the AUDIT was administered and, hence, were included in the current analyses. The median number of completed follow-up surveys that included the AUDIT was 4 surveys (range, 1-6 surveys; interquartile range, 2-5 surveys).

Over the 6-year survey period, according to the full AUDIT, 18 577 participants (82.7%) were abstinent or had a score of less than 8, 1909 participants (8.5%) had a score of 8 to 15, 363 participants (1.6%) had a score of 16 to 19, and 671 participants (3.0%) had a score of 20 to 40 (Table 1). On the basis of the AUDIT-C, 17 321 participants (77.1%) had a score of less than 4, 2659 participants (11.8%) had a score of 4 to 5, 1234 participants (5.5%) had a score of 6 to 7, and 1009 participants (4.5%) had a score of 8 or higher. The percentage in each risk category of the AUDIT or AUDIT-C remained generally stable over time.

**Table 1.** Across-Time Prevalence of Alcohol Use Severity, Psychiatric Disorder Symptoms, and Substance Use Among Veterans Aging Cohort Study Participants, 2003 to 2012^a^

Alcohol-Clustering Condition	Participants, No. (%)						
	Survey Wave						Overall (N = 6431)^b^
	2003-2004 (n = 2883)	2004-2005 (n = 3997)	2005-2007 (n = 4112)	2008-2009 (n = 4252)	2009-2011 (n = 3764)	2011-2012 (n = 3515)	
Alcohol Use Disorders Identification Test score							
<8	2380 (84.0)	2989 (74.8)	3412 (83.0)	3565 (83.8)	3203 (85.1)	3028 (86.2)	18 577 (82.7)
8-15	214 (7.6)	333 (8.3)	411 (10.0)	394 (9.3)	290 (7.7)	267 (7.6)	1909 (8.5)
16-19	46 (1.6)	70 (1.8)	66 (1.6)	71 (1.7)	64 (1.7)	46 (1.3)	363 (1.6)
20-40	74 (2.6)	138 (3.5)	146 (3.6)	122 (2.9)	104 (2.8)	87 (2.5)	671 (3.0)
Alcohol Use Disorders Identification Test–Consumption score							
<4	2129 (75.2)	3086 (77.2)	3126 (76.0)	3274 (77.0)	2896 (76.9)	2810 (79.9)	17 321 (77.1)
4-5	382 (13.5)	477 (11.9)	482 (11.7)	491 (11.6)	462 (12.3)	365 (10.4)	2659 (11.8)
6-7	151 (5.3)	233 (5.8)	240 (5.8)	240 (5.6)	206 (5.5)	164 (4.7)	1234 (5.5)
≥8	124 (4.4)	183 (4.6)	231 (5.6)	194 (4.6)	147 (3.9)	130 (3.7)	1009 (4.5)
Psychiatric symptoms							
Depression	553 (20.3)	917 (23.1)	1035 (25.4)	851 (20.2)	755 (20.3)	752 (21.6)	4863 (21.9)
Anxiety	1196 (45.2)	1832 (46.7)	1856 (46.1)	1937 (46.5)	NA^c^	1464 (42.8)	8285 (45.6)
Drug use							
Tobacco	NA^c^	1118 (32.1)	NA^c^	1113 (26.6)	896 (24.2)	815 (23.4)	3942 (26.5)
Marijuana	549 (20.3)	826 (20.7)	816 (20.7)	770 (18.8)	716 (19.6)	667 (19.5)	4344 (19.9)
Crack or cocaine	287 (10.6)	633 (15.8)	616 (15.7)	551 (13.5)	470 (13.0)	370 (10.9)	2927 (13.5)
Stimulants other than crack or cocaine^d^	57 (2.1)	120 (3.0)	99 (2.5)	87 (2.1)	66 (1.8)	61 (1.8)	490 (2.3)
Illicit opioids^e^	449 (16.4)	621 (20.1)	187 (4.8)	707 (17.2)	607 (16.6)	652 (19.0)	3223 (15.4)
Injection drugs	56 (2.0)	113 (2.8)	118 (2.9)	120 (2.9)	96 (2.6)	95 (2.7)	598 (2.7)

Abbreviation: NA, not applicable.

^a^ Totals may not sum to the number participating in each survey wave because of missing values.

^b^ Prevalence values are based on responses of 6431 Veterans Aging Cohort Study respondents who participated in at least 1 survey over the follow-up period (22 473 surveys total).

^c^ Data were not assessed at this survey wave.

^d^ Past-year use of stimulants, defined as amphetamines, uppers, speed, crank, crystal meth, or bam.

^e^ Includes use of prescription opioids or painkillers (eg, oxycodone or hydrocodone) or heroin use. Prescription opioids were not assessed during the 2005 to 2007 survey wave.

Over the follow-up period, 4863 respondents (21.9%) reported depressive symptoms and 8285 (45.6%) had anxiety symptoms. In the past year, more than one-quarter of the sample had substantial tobacco use (3942 participants [26.5%]), 4344 participants (19.9%) reported using marijuana, 2927 participants (13.5%) reported crack or cocaine use, and 3223 participants (15.4%) reported illicit opioid use. A minority of the sample reported using stimulants other than crack or cocaine (490 participants [2.3%]) and injection drugs (598 participants [2.7%]). The prevalence of psychiatric disorder symptoms and substance use varied over time.

The analytical sample of 6431 participants was comparable with the 1079 individuals who were omitted from the analysis, including with regard to age (median age, 50 years in both groups), sex (95% male in both groups), black and Hispanic race/ethnicity (approximately 75% in both groups), and having an annual income of less than $12 000 (48% baseline only; 48% follow-up sample), as well as with regard to unhealthy alcohol use defined as an AUDIT-C score of 4 or higher (39% baseline only; 37% follow-up sample), depression (22% baseline only; 20% follow-up sample), anxiety (44% baseline only; 45% follow-up sample), marijuana use (21% baseline only; 23% follow-up sample), and cocaine use (19% in both groups).

### Alcohol Use Patterns and Alcohol-Clustering Conditions

We observed a general dose-response association between alcohol use severity category and clustering condition when using the AUDIT or the AUDIT-C to assess alcohol use. The ORs for the associations between AUDIT scores of 8 to 15, 16 to 19, and 20 to 40 vs 0 to 7, the reference, ranged from 1.90 (95% CI, 1.58-2.29) to 8.37 (95% CI, 6.20-11.29) for depression and 2.07 (95% CI, 1.73-2.49) to 8.98 (95% CI, 6.39-12.60) for anxiety symptoms (Table 2). The ORs were larger when alcohol use was assessed using the AUDIT vs the AUDIT-C. The highest category of the AUDIT (≥20 vs <8, the reference) was associated with greater than 10 times odds of using tobacco (OR, 14.64, 95% CI, 8.94-23.98), crack or cocaine (OR, 39.47, 95% CI, 27.38-56.90), stimulants other than crack or cocaine (OR, 21.31, 95% CI, 12.73-35.67), marijuana (OR, 12.41; 95% CI, 8.61-17.90), and injection drugs (OR, 8.67; 95% CI, 5.32-14.13). An AUDIT score of 20 or higher yielded likelihood ratio values greater than 3.5 for depression, anxiety, crack or cocaine use, and other stimulant use. Associations did not vary significantly by HIV status (results not shown); hence, the findings are presented for the full sample.

**Table 2.** Associations Between Alcohol Use Severity, Psychiatric Disorder Symptoms, and Substance Use Among Veterans Aging Cohort Study Participants

Alcohol-Clustering Condition	Unadjusted OR (95% CI)					
	Alcohol Use Disorders Identification Test^a^			Alcohol Use Disorders Identification Test–Consumption^b^		
	Score ≥8	Score ≥16	Score ≥20	Score ≥4	Score ≥6	Score ≥8
Depression	1.90 (1.58-2.29)	3.71 (2.58-5.35)	8.37 (6.20-11.29)	1.29 (1.10-1.53)	1.55 (1.24-1.94)	2.78 (2.18-3.55)
Anxiety	2.07 (1.73-2.49)	2.42 (1.66-3.52)	8.98 (6.39-12.60)	1.42 (1.22-1.66)	1.50 (1.21-1.87)	2.06 (1.60-2.64)
Tobacco^c^	4.60 (3.44-6.16)	3.38 (1.90-6.02)	14.64 (8.94-23.98)	2.66 (2.07-3.41)	5.48 (3.89-7.72)	12.71 (8.39-19.24)
Marijuana	3.37 (2.70-4.22)	6.48 (4.22-9.93)	12.41 (8.61-17.90)	3.77 (3.09-4.58)	4.50 (3.47-5.84)	6.78 (5.01-9.16)
Crack or cocaine	6.55 (5.23-8.20)	12.07 (7.86-18.54)	39.47 (27.38-56.90)	4.00 (3.25-4.93)	9.66 (7.40-12.60)	13.51 (10.01-18.24)
Stimulants other than crack or cocaine^d^	3.98 (2.62-6.04)	4.39 (2.00-9.62)	21.31 (12.73-35.67)	2.14 (1.47-3.13)	2.77 (1.69 -4.54)	8.14 (5.00-13.25)
Illicit opioid^e^	1.24 (1.03-1.50)	2.35 (1.64-3.36)	2.35 (1.76-3.15)	1.00 (0.85-1.19)	1.03 (0.82-1.30)	1.49 (1.16-1.92)
Injection drugs	2.44 (1.69-3.52)	2.53 (1.25-5.12)	8.67 (5.32-14.13)	2.07 (1.46-2.93)	2.61 (1.65-4.12)	2.95 (1.79-4.86)

Abbreviation: OR, odds ratio.

^a^ Alcohol Use Disorders Identification Test scores range from 0 to 40, with a score of 40 indicating the highest severity level of alcohol use. These analyses used a score of less than 8 as the reference group.

^b^ Alcohol Use Disorders Identification Test–Consumption scores range from 0 to 12, with a score of 12 indicating the highest level of alcohol use. These analyses used a score of less than 4 as the reference group.

^c^ Defined as 10 or more cigarettes per day.

^d^ Includes amphetamines, uppers, speed, crank, crystal methamphetamine, and bam.

^e^ Includes use of prescription opioids or painkillers (eg, oxycodone or hydrocodone) or heroin use. Prescription opioids were not assessed during the 2005 to 2007 survey wave.

### Test Performance of Alcohol Use Screening for Indication of Alcohol-Clustering Conditions

#### Alcohol Use to Identify Cases of Depression and Anxiety

An AUDIT score of 8 or above was 21.4% sensitive and 85.5% specific for depressive symptoms, with a PPV of 34.2% and likelihood ratio of 1.86; for anxiety symptoms, an AUDIT score of 8 or higher was 18.7% sensitive and 90.0% specific, with a PPV of 61.1% and a likelihood ratio of 1.87 (Table 3). An AUDIT score of 16 or higher was 10.0% sensitive and 96.7% specific for depressive symptoms, with a PPV of 45.7% and likelihood ratio of 3.00; for anxiety symptoms, an AUDIT score of 16 or higher was 7.5% sensitive and 97.4% specific, with a PPV of 70.5% and a likelihood ratio of 2.84 (Table 3). An AUDIT score of 20 or higher was 7.2% sensitive and 98.0% specific for depressive symptoms, with a PPV of 50.4% and a likelihood ratio of 3.63; for anxiety symptoms, an AUDIT score of 20 or higher was 5.3% sensitive and 98.6% specific, with a PPV of 76.6% and a likelihood ratio of 3.90. When using an AUDIT score cut point of 20 or greater, the likelihood ratios for detection of depression approached that of the PHQ-2 (AUDIT, 3.63; PHQ-2, 4.0) and those for anxiety approached that of the GAD-7 (AUDIT, 3.90; GAD-7, 5.1) (Figure). Categorization of alcohol use severity based on the AUDIT-C yielded slight increases in sensitivity but reduced specificity and PPVs.

**Table 3.** Test Performance of Alcohol Use Severity Indicators for Indication of Psychiatric Disorder Symptoms and Substance Use Among Veterans Aging Cohort Study Participants

Alcohol-Clustering Condition	Percentage					
	Alcohol Use Disorders Identification Test			Alcohol Use Disorders Identification Test–Consumption		
	Score ≥8	Score ≥16	Score ≥20	Score ≥4	Score ≥6	Score ≥8
Depression						
Sensitivity	21.4	10.0	7.2	26.1	13.8	7.4
Specificity	85.5	96.7	98.0	79.1	91.2	96.3
Positive predictive value	34.2	45.7	50.4	25.9	30.0	36.0
Likelihood ratio	1.86	3.00	3.63	1.24	1.53	2.00
Correctly classified	73.8	77.7	78.2	67.4	74.2	76.8
Anxiety						
Sensitivity	18.7	7.5	5.3	24.7	12.1	5.9
Specificity	90.0	97.4	98.6	80.2	91.4	96.4
Positive predictive value	61.1	70.5	76.6	51.1	54.0	57.7
Likelihood ratio	1.87	2.84	3.90	1.25	1.40	1.63
Correctly classified	57.5	56.4	56.1	54.9	55.2	55.1
Tobacco use (≥10 cigarettes/d)						
Sensitivity	22.6	9.0	6.1	30.9	17.2	8.2
Specificity	89.7	96.8	98.1	81.6	92.7	97.1
Positive predictive value	43.9	49.8	52.9	37.8	46.0	50.6
Likelihood ratio	2.18	2.76	3.13	1.69	2.42	2.84
Correctly classified	72.0	73.6	73.8	68.2	72.7	73.6
Marijuana use						
Sensitivity	23.5	9.3	6.3	35.4	17.2	7.8
Specificity	88.8	96.4	98.7	81.2	91.7	96.3
Positive predictive value	34.2	38.9	40.1	32.0	34.2	34.6
Likelihood ratio	2.10	2.56	2.70	1.89	2.08	2.12
Correctly classified	75.8	79.1	79.5	72.1	76.9	78.7
Crack or cocaine use						
Sensitivity	36.0	16.6	11.5	42.6	25.1	11.9
Specificity	89.7	97.0	98.2	81.1	92.3	96.6
Positive predictive value	35.1	46.1	49.2	25.9	33.5	35.4
Likelihood ratio	3.51	5.56	6.27	2.25	3.23	3.52
Correctly classified	82.6	86.3	86.6	75.9	83.4	85.2
Stimulants other than crack or cocaine^a^						
Sensitivity	32.7	17.6	14.4	38.0	21.4	12.7
Specificity	86.9	95.6	97.2	78.4	90.3	95.7
Positive predictive value	5.6	8.4	10.6	3.9	4.9	6.5
Likelihood ratio	2.57	3.99	5.16	1.76	2.20	2.99
Correctly classified	85.7	93.8	95.4	77.5	88.7	93.9
Illicit opioids^b^						
Sensitivity	17.3	7.4	4.7	23.5	11.3	5.6
Specificity	86.7	95.6	97.1	77.7	89.9	95.6
Positive predictive value	19.1	23.2	23.0	16.1	17.0	19.0
Likelihood ratio	1.30	1.66	1.65	1.05	1.12	1.28
Correctly classified	76.1	82.1	83.0	69.4	77.8	81.7
Injection drugs						
Sensitivity	30.4	14.0	10.6	34.3	17.5	8.4
Specificity	86.8	95.4	97.1	78.3	90.1	95.6
Positive predictive value	5.9	7.8	9.0	4.2	4.7	5.0
Likelihood ratio	2.30	3.07	3.59	1.58	1.77	1.89
Correctly classified	85.2	93.3	94.8	77.1	88.1	93.2

^a^ Includes amphetamines, uppers, speed, crank, crystal methamphetamine, or bam.

^b^ Includes use of prescription opioids or painkillers (eg, oxycodone or hydrocodone) or heroin use. Prescription opioids were not assessed during the 2005 to 2007 survey wave.

**Figure.**
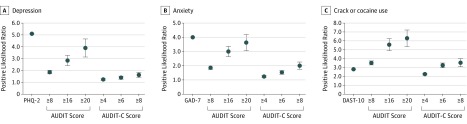
Test Performance of Alcohol Screening Scores for Identification of Depression, Anxiety, and Crack or Cocaine Use A, Values are shown for the Patient Health Questionnaire–2 (PHQ-2) vs Alcohol Use Disorders Identification Test (AUDIT) and Alcohol Use Disorders Identification Test–Consumption (AUDIT-C) for detection of depression. B, Values are shown for the Generalized Anxiety Disorder 7-Item Scale (GAD-7) vs AUDIT and AUDIT-C for detection of anxiety. C, Values are shown for the Drug Abuse Screen Test (DAST-10) vs AUDIT and AUDIT-C for detection of crack or cocaine use. Error bars denote 95% CIs.

#### Alcohol Use to Identify Cases of Other Substance Use

An AUDIT score of 8 or higher yielded sensitivity levels of 17.3% for indication of illicit opioid use, 22.6% for substantial tobacco use, 23.5% for marijuana use, 30.4% for injection drugs, 36.0% for crack or cocaine use, and 32.7% for use of other stimulants, with specificities of 86.7% or higher for indication of each substance use outcome (Table 3). The PPVs were greatest for indication of marijuana (34.2%), crack or cocaine (35.1%), and substantial tobacco use (43.9%) and were much lower for illicit opioid use (19.1%), other stimulant use (5.6%), and injection drug use (5.9%). Likelihood ratios ranged from 1.30 (illicit opioid use) to 3.51 (crack or cocaine). When a positive screen was defined using AUDIT score thresholds of 16 or higher and 20 or higher, sensitivity decreased, whereas specificity, PPVs, and likelihood ratios increased. The AUDIT appeared to yield much higher likelihood ratio values for the detection of crack or cocaine use than those estimated for the DAST-10 (likelihood ratios: AUDIT score ≥8, 3.51; AUDIT score ≥16, 5.56; AUDIT score ≥20, 6.27 vs DAST-10, 2.8). For an AUDIT score of 20 or higher, the PPV for the detection of crack or cocaine use was 49.2%.

Screening based on the AUDIT-C vs the AUDIT resulted in slight increases in sensitivity, reductions in specificity, and decreases in PPV and likelihood ratios. The likelihood ratios for the detection of crack or cocaine use when using the AUDIT-C approached or exceeded the likelihood ratios when using the DAST-10 (likelihood ratios: AUDIT-C score ≥4, 2.25; AUDIT score ≥6, 3.23; AUDIT score ≥8, 3.52 vs DAST-10, 2.8) (Figure).

## Discussion

This study is the first, to our knowledge, to assess the value of using the AUDIT and AUDIT-C for the potential identification of conditions that commonly cluster with alcohol use. Our results raise the question of whether using the AUDIT or AUDIT-C to screen for unhealthy alcohol use in primary care contains enough incidental information about the likelihood of alcohol-clustering conditions to affect screening decisions for these other conditions. For example, an AUDIT score of 20 or higher yielded likelihood ratio values greater than 3.5 for depression, anxiety, and crack or cocaine and other stimulant use. In a sufficiently high prevalence population, these likelihood ratios may confer a PPV sufficiently high to merit a diagnostic assessment for anxiety. Even in lower prevalence populations, these likelihood ratios may be sufficiently high to cause a clinically meaningful elevation of the PPV of anxiety screening, potentially making anxiety screening more clinically useful. Although the AUDIT and the AUDIT-C had low-to-moderate sensitivity for detecting alcohol-clustering conditions, their moderate-to-high likelihood ratio values and PPVs show they convey substantial information regarding the likely presence of these conditions. As long as the AUDIT or AUDIT-C are being administered anyway for alcohol screening, this additional information may be sufficient to newly motivate screening or definitive diagnostic efforts for alcohol-clustering conditions.

For example, among VACS enrollees scoring in the highest AUDIT category (AUDIT score, 20-40), 76.6% would screen positive for anxiety symptoms, 50.4% would screen positive for depressive symptoms, and 49.2% would screen positive for crack or cocaine use. The AUDIT and AUDIT-C also had high-to-excellent levels of specificity, which yielded high percentages of individuals correctly classified and likelihood ratio values that are comparable with those of dedicated screeners. Alcohol screening had likelihood ratio values that approached those of the GAD-7^46^ for indication of anxiety and better likelihood ratio values than the DAST-10^47^ for indication of crack or cocaine use.

These findings suggest a need for decision analytic modeling to systematically weigh the advantages vs the disadvantages of using the AUDIT to guide use of screeners for other conditions. Our results also reinvigorate the question of whether use of the full AUDIT compared with the AUDIT-C contains sufficient additional information to be worth the added response burden and imposition on clinical workflow.

Our findings that scores on 2 widely used alcohol screening tools are associated with anxiety symptoms depressive symptoms and other substance use corroborate those from prior studies^17,18,19,20,21,22,23,24,53,54,55,56,57,58,59,60,61^ and are consistent with neuroscientific findings regarding reward circuitry pathways in the brain and what is known about the genetics of alcohol, substance use, and mental health conditions. No guidelines currently recommend that identification of unhealthy alcohol use should prompt screening for alcohol-clustering conditions. If corroborated by future studies, our results suggest that guideline panels should consider whether an expanded scope for the AUDIT and AUDIT-C is warranted given their utility in informing the index of suspicion for other alcohol-clustering conditions. There is precedent for using screening for a particular condition to improve case finding for related and/or clustering conditions. For example, in the context of clinical management of sexually transmitted infection, identification and treatment of gonorrhea would lead to treatment of chlamydia even in the absence of biological confirmation of chlamydial infection.^62^ Furthermore, our findings reinforce the importance of promoting evidence-based screening in routine medical settings, which currently are not used consistently.^63,64,65^

The full AUDIT demonstrated better overall test performance indicated by greater likelihood ratio values and slightly higher percentages of individuals correctly classified compared with the AUDIT-C. Accordingly, the full AUDIT, despite its greater length, may be preferable to the AUDIT-C as a tool in clinical practice, given the additional benefit of identifying those at high risk of psychiatric disorders and other substance use in addition to identifying those with unhealthy alcohol use.

### Limitations

This study has some limitations that should be noted. Most importantly, we assessed the presence of alcohol-clustering conditions using brief screening tools (ie, PHQ) or self-reported endorsement (eg, anxiety symptoms or drug use) rather than diagnoses using a formal instrument. It is possible the AUDIT or the AUDIT-C would have different associations with clinically diagnosed conditions; hence, our findings on test performance of these tools for identification of conditions would be affected. Another important limitation is that study findings are only generalizable to veterans receiving care in the Veterans Health Administration. It is possible that associations between unhealthy alcohol use and other conditions may differ among veterans compared with nonveterans. Additional studies are hence needed to assess alcohol use screening as an indicator of associated conditions across diverse samples. A goal of the present study was to assess whether evidence-based cut points indicating unhealthy alcohol use may also serve to guide screening for comorbid conditions. Future studies should explore continuous alcohol use indicators, in which a range of alcohol score values are assessed for indication of clustering conditions.

## Conclusions

Our findings underscore the potential for alcohol screening, which is recommended as a standard practice in most primary care settings, to provide an additional benefit of identifying patients with a high risk of other clinical conditions. Using information from alcohol screening to trigger assessment of conditions expected to cluster with alcohol use appears to be a promising way to improve case finding and, by extension, treatment of depression, anxiety, and drug use disorder. Additional studies in other populations will provide insight into the degree to which alcohol screening is useful for identification of alcohol-clustering conditions across populations. In addition, assessment of the degree to which other conditions or behaviors that are commonly assessed in clinical practice (eg, tobacco use) can help improve case finding and treatment of important health concerns is warranted.

## References

[1] Grant BF, Chou SP, Saha TD, Prevalence of 12-month alcohol use, high-risk drinking, and *DSM-IV* alcohol use disorder in the United States, 2001-2002 to 2012-2013: results from the national epidemiologic survey on alcohol and related conditions. JAMA Psychiatry. 2017;74(9):-. doi:10.1001/jamapsychiatry.2017.2161 28793133 PMC5710229

[2] Degenhardt L, Charlson F, Ferrari A, ; GBD 2016 Alcohol and Drug Use Collaborators The global burden of disease attributable to alcohol and drug use in 195 countries and territories, 1990-2016: a systematic analysis for the Global Burden of Disease Study 2016. Lancet Psychiatry. 2018;5(12):987-1012. doi:10.1016/S2215-0366(18)30337-7 30392731 PMC6251968

[3] US Department of Health and Human Services and US Department of Agriculture Dietary guidelines for Americans, 2015-2020: 8th edition. Published December 2015 Accessed June 13, 2019. https://health.gov/dietaryguidelines/2015/guidelines/

[4] Saitz R Clinical practice: unhealthy alcohol use. N Engl J Med. 2005;352(6):596-607. doi:10.1056/NEJMcp042262 15703424

[5] Centers for Disease Control and Prevention Planning and Implementing Screening and Brief Intervention for Risky Alcohol Use: A Step-by-Step Guide for Primary Care Practices. Centers for Disease Control and Prevention, National Center on Birth Defects and Developmental Disabilities; 2014.

[6] Curry SJ, Krist AH, Owens DK, ; US Preventive Services Task Force Screening and behavioral counseling interventions to reduce unhealthy alcohol use in adolescents and adults: US Preventive Services Task Force recommendation statement. JAMA. 2018;320(18):1899-1909. doi:10.1001/jama.2018.16789 30422199

[7] Bachrach RL, Blosnich JR, Williams EC Alcohol screening and brief intervention in a representative sample of veterans receiving primary care services. J Subst Abuse Treat. 2018;95:18-25. doi:10.1016/j.jsat.2018.09.003 30352666 PMC6211809

[8] Substance Abuse and Mental Health Services Administration SBIRT: a resource toolkit for behavioral health providers to begin the conversation with federally qualified healthcare centers. Published 2019 Accessed June 13, 2019. http://www.integration.samhsa.gov/sbirt_toolkit_for_working_with_fqhcs.pdf

[9] Kessler RC, McGonagle KA, Zhao S, Lifetime and 12-month prevalence of *DSM-III-R* psychiatric disorders in the United States: results from the National Comorbidity Survey. Arch Gen Psychiatry. 1994;51(1):8-19. doi:10.1001/archpsyc.1994.03950010008002 8279933

[10] Kessler RC, Berglund P, Demler O, Jin R, Merikangas KR, Walters EE Lifetime prevalence and age-of-onset distributions of *DSM-IV* disorders in the National Comorbidity Survey Replication. Arch Gen Psychiatry. 2005;62(6):593-602. doi:10.1001/archpsyc.62.6.593 15939837

[11] Centers for Disease Control and Prevention Illicit drug use. Published 2017 Accessed March 11, 2019. https://www.cdc.gov/nchs/fastats/drug-use-illegal.htm

[12] US Preventive Services Task Force USPSTF A and B recommendations. Published 2019 Accessed March 11, 2019. https://www.uspreventiveservicestaskforce.org/Page/Name/uspstf-a-and-b-recommendations/

[13] McCormick KA, Cochran NE, Back AL, Merrill JO, Williams EC, Bradley KA How primary care providers talk to patients about alcohol: a qualitative study. J Gen Intern Med. 2006;21(9):966-972. doi:10.1007/BF02743146 16918743 PMC1831591

[14] Williams EC, Achtmeyer CE, Young JP, Local implementation of alcohol screening and brief intervention at five Veterans Health Administration primary care clinics: perspectives of clinical and administrative staff. J Subst Abuse Treat. 2016;60:27-35. doi:10.1016/j.jsat.2015.07.011 26297322

[15] Kushner MG, Abrams K, Borchardt C The relationship between anxiety disorders and alcohol use disorders: a review of major perspectives and findings. Clin Psychol Rev. 2000;20(2):149-171. doi:10.1016/S0272-7358(99)00027-6 10721495

[16] Boden JM, Fergusson DM Alcohol and depression. Addiction. 2011;106(5):906-914. doi:10.1111/j.1360-0443.2010.03351.x 21382111

[17] Substance Abuse and Mental Health Services Administration Results From the 2012 National Survey on Drug Use and Health: Summary of National Findings. Substance Abuse and Mental Health Services Administration; 2013.

[18] Reed MB, Wang R, Shillington AM, Clapp JD, Lange JE The relationship between alcohol use and cigarette smoking in a sample of undergraduate college students. Addict Behav. 2007;32(3):449-464. doi:10.1016/j.addbeh.2006.05.016 16844313

[19] McCabe SE, Cranford JA, Boyd CJ The relationship between past-year drinking behaviors and nonmedical use of prescription drugs: prevalence of co-occurrence in a national sample. Drug Alcohol Depend. 2006;84(3):281-288. doi:10.1016/j.drugalcdep.2006.03.006 16621337 PMC1706074

[20] Anglin MD, Almog IJ, Fisher DG, Peters KR Alcohol use by heroin addicts: evidence for an inverse relationship—a study of methadone maintenance and drug-free treatment samples. Am J Drug Alcohol Abuse. 1989;15(2):191-207. doi:10.3109/00952998909092720 2729226

[21] Welte JW, Barnes GM The relationship between alcohol use and other drug use among New York State college students. Drug Alcohol Depend. 1982;9(3):191-199. doi:10.1016/0376-8716(82)90044-8 6981498

[22] Substance Abuse and Mental Health Services Administration National Survey on Drug Use and Health. Substance Abuse and Mental Health Services Administration; 2014.

[23] Gossop M, Manning V, Ridge G Concurrent use and order of use of cocaine and alcohol: behavioural differences between users of crack cocaine and cocaine powder. Addiction. 2006;101(9):1292-1298. doi:10.1111/j.1360-0443.2006.01497.x 16911728

[24] Marks KR, Pike E, Stoops WW, Rush CR Alcohol administration increases cocaine craving but not cocaine cue attentional bias. Alcohol Clin Exp Res. 2015;39(9):1823-1831. doi:10.1111/acer.12824 26331880 PMC4562057

[25] Smith PC, Cheng DM, Allensworth-Davies D, Winter MR, Saitz R Use of a single alcohol screening question to identify other drug use. Drug Alcohol Depend. 2014;139:178-180. doi:10.1016/j.drugalcdep.2014.03.027 24768061 PMC4085274

[26] Spirito A, Bromberg JR, Casper TC, ; Pediatric Emergency Care Research Network (PECARN) Screening for adolescent alcohol use in the emergency department: what does it tell us about cannabis, tobacco, and other drug use? Subst Use Misuse. 2019;54(6):1007-1016. doi:10.1080/10826084.2018.1558251 30727811 PMC6476662

[27] Babor TF, Higgins-Biddle JC, Saunders JB, Monteiro MG The Alcohol Use Disorders Identification Test: Guidelines for Use in Primary Care. 2nd ed World Health Organization; 2001.

[28] Osaki Y, Ino A, Matsushita S, Higuchi S, Kondo Y, Kinjo A Reliability and validity of the alcohol use disorders identification test: consumption in screening for adults with alcohol use disorders and risky drinking in Japan. Asian Pac J Cancer Prev. 2014;15(16):6571-6574. doi:10.7314/APJCP.2014.15.16.6571 25169489

[29] Khadjesari Z, White IR, McCambridge J, Validation of the AUDIT-C in adults seeking help with their drinking online. Addict Sci Clin Prac. 2017;12(1):2. doi:10.1186/s13722-016-0066-5 28049515 PMC5209877

[30] Frank D, DeBenedetti AF, Volk RJ, Williams EC, Kivlahan DR, Bradley KA Effectiveness of the AUDIT-C as a screening test for alcohol misuse in three race/ethnic groups. J Gen Intern Med. 2008;23(6):781-787. doi:10.1007/s11606-008-0594-0 18421511 PMC2517893

[31] Yarnall KSH, Pollak KI, Østbye T, Krause KM, Michener JL Primary care: is there enough time for prevention? Am J Public Health. 2003;93(4):635-641. doi:10.2105/AJPH.93.4.635 12660210 PMC1447803

[32] Justice AC, Dombrowski E, Conigliaro J, Veterans Aging Cohort Study (VACS): overview and description. Med Care. 2006;44(8)(suppl 2):S13-S24. doi:10.1097/01.mlr.0000223741.02074.66 16849964 PMC3049942

[33] Gache P, Michaud P, Landry U, The Alcohol Use Disorders Identification Test (AUDIT) as a screening tool for excessive drinking in primary care: reliability and validity of a French version. Alcohol Clin Exp Res. 2005;29(11):2001-2007. doi:10.1097/01.alc.0000187034.58955.64 16340457

[34] Adewuya AO Validation of the Alcohol Use Disorders Identification Test (AUDIT) as a screening tool for alcohol-related problems among Nigerian university students. Alcohol. 2005;40(6):575-577. doi:10.1093/alcalc/agh197 16115823

[35] Davey JD, Obst PL, Sheehan MC The use of AUDIT as a screening tool for alcohol use in the police work-place. Drug Alcohol Rev. 2000;19(1):49-54. doi:10.1080/09595230096147

[36] Marshall BDL, Tate JP, McGinnis KA, Long-term alcohol use patterns and HIV disease severity. AIDS. 2017;31(9):1313-1321. doi:10.1097/QAD.0000000000001473 28492393 PMC5596310

[37] Bradley KA, DeBenedetti AF, Volk RJ, Williams EC, Frank D, Kivlahan DR AUDIT-C as a brief screen for alcohol misuse in primary care. Alcohol Clin Exp Res. 2007;31(7):1208-1217. doi:10.1111/j.1530-0277.2007.00403.x 17451397

[38] Williams EC, Bryson CL, Sun H, Association between alcohol screening results and hospitalizations for trauma in Veterans Affairs outpatients. Am J Drug Alcohol Abuse. 2012;38(1):73-80. doi:10.3109/00952990.2011.600392 21797815

[39] Manea L, Gilbody S, McMillan D Optimal cut-off score for diagnosing depression with the Patient Health Questionnaire (PHQ-9): a meta-analysis. CMAJ. 2012;184(3):E191-E196. doi:10.1503/cmaj.110829 22184363 PMC3281183

[40] Kroenke K, Spitzer RL, Williams JB The PHQ-9: validity of a brief depression severity measure. J Gen Intern Med. 2001;16(9):606-613. doi:10.1046/j.1525-1497.2001.016009606.x 11556941 PMC1495268

[41] Arroll B, Goodyear-Smith F, Crengle S, Validation of PHQ-2 and PHQ-9 to screen for major depression in the primary care population. Ann Fam Med. 2010;8(4):348-353. doi:10.1370/afm.1139 20644190 PMC2906530

[42] Justice AC, Holmes W, Gifford AL, ; Adult AIDS Clinical Trials Unit Outcomes Committee Development and validation of a self-completed HIV symptom index. J Clin Epidemiol. 2001;54(1)(suppl):S77-S90. doi:10.1016/S0895-4356(01)00449-8 11750213

[43] Edelman EJ, Gordon K, Rodriguez-Barradas MC, Justice AC; VACS Project Team Patient-reported symptoms on the antiretroviral regimen efavirenz/emtricitabine/tenofovir. AIDS Patient Care STDS. 2012;26(6):312-319. doi:10.1089/apc.2012.0044 22612469 PMC3412583

[44] Young Q-R, Nguyen M, Roth S, Broadberry A, Mackay MH Single-item measures for depression and anxiety: validation of the screening tool for psychological distress in an inpatient cardiology setting. Eur J Cardiovasc Nurs. 2015;14(6):544-551. doi:10.1177/1474515114548649 25139467

[45] Spitzer RL, Kroenke K, Williams JBW, Löwe B A brief measure for assessing generalized anxiety disorder: the GAD-7. Arch Intern Med. 2006;166(10):1092-1097. doi:10.1001/archinte.166.10.1092 16717171

[46] Herr NR, Williams JW Jr, Benjamin S, McDuffie J Does this patient have generalized anxiety or panic disorder? the Rational Clinical Examination systematic review. JAMA. 2014;312(1):78-84. doi:10.1001/jama.2014.5950 25058220

[47] Villalobos-Gallegos L, Perez-Lopez A, Mendoza-Hassey R, Graue-Moreno J, Marin-Navarrete R Psychometric and diagnostic properties of the Drug Abuse Screening Test (DAST): comparing the DAST-20 vs. the DAST-10. Salud Ment. 2015;38(2):89-94. doi:10.17711/SM.0185-3325.2015.012

[48] Skinner HA The drug abuse screening test. Addict Behav. 1982;7(4):363-371. doi:10.1016/0306-4603(82)90005-3 7183189

[49] Koepsell TD, Martin DC, Diehr PH, Data analysis and sample size issues in evaluations of community-based health promotion and disease prevention programs: a mixed-model analysis of variance approach. J Clin Epidemiol. 1991;44(7):701-713. doi:10.1016/0895-4356(91)90030-D 2066748

[50] Salonen JT, Kottke TE, Jacobs DR Jr, Hannan PJ Analysis of community-based cardiovascular disease prevention studies: evaluation issues in the North Karelia Project and the Minnesota Heart Health Program. Int J Epidemiol. 1986;15(2):176-182. doi:10.1093/ije/15.2.176 3721679

[51] Thompson DR A randomized controlled trial of in-hospital nursing support for first time myocardial infarction patients and their partners: effects on anxiety and depression. J Adv Nurs. 1989;14(4):291-297. doi:10.1111/j.1365-2648.1989.tb03416.x 2738227

[52] Zhou H, Weinberg CR Potential for bias in estimating human fecundability parameters: a comparison of statistical models. Stat Med. 1999;18(4):411-422. doi:10.1002/(SICI)1097-0258(19990228)18:4<411::AID-SIM26>3.0.CO;2-M 10070683

[53] Jones-Webb R, Jacobs DR Jr, Flack JM, Liu K. Relationships between depressive symptoms, anxiety, alcohol consumption, and blood pressure: results from the CARDIA study. Alcohol Clin Exp Res. 1996;20(3):420-427. doi:10.1111/j.1530-0277.1996.tb01069.x 8727231

[54] Kaplow JB, Curran PJ, Angold A, Costello EJ The prospective relation between dimensions of anxiety and the initiation of adolescent alcohol use. J Clin Child Psychol. 2001;30(3):316-326. doi:10.1207/S15374424JCCP3003_4 11501249

[55] Awaworyi Churchill S, Farrell L Alcohol and depression: evidence from the 2014 health survey for England. Drug Alcohol Depend. 2017;180:86-92. doi:10.1016/j.drugalcdep.2017.08.006 28886396

[56] Smith JP, Randall CL Anxiety and alcohol use disorders: comorbidity and treatment considerations. Alcohol Res. 2012;34(4):414-431. 23584108 10.35946/arcr.v34.4.06 PMC3860396

[57] Partonen T Clock genes in human alcohol abuse and comorbid conditions. Alcohol. 2015;49(4):359-365. doi:10.1016/j.alcohol.2014.08.013 25677407

[58] Sullivan LE, Goulet JL, Justice AC, Fiellin DA Alcohol consumption and depressive symptoms over time: a longitudinal study of patients with and without HIV infection. Drug Alcohol Depend. 2011;117(2-3):158-163. doi:10.1016/j.drugalcdep.2011.01.014 21345624 PMC3113463

[59] Sullivan LE, Fiellin DA, O’Connor PG The prevalence and impact of alcohol problems in major depression: a systematic review. Am J Med. 2005;118(4):330-341. doi:10.1016/j.amjmed.2005.01.007 15808128

[60] Grant BF, Harford TC Comorbidity between *DSM-IV* alcohol use disorders and major depression: results of a national survey. Drug Alcohol Depend. 1995;39(3):197-206. doi:10.1016/0376-8716(95)01160-4 8556968

[61] Grant BF, Hasin DS, Dawson DA The relationship between DSM-IV alcohol use disorders and *DSM-IV* major depression: examination of the primary-secondary distinction in a general population sample. J Affect Disord. 1996;38(2-3):113-128. doi:10.1016/0165-0327(96)00002-X 8791180

[62] Workowski KA, Bolan GA; Centers for Disease Control and Prevention Sexually transmitted diseases treatment guidelines, 2015. MMWR Recomm Rep. 2015;64(RR-03):1-137. 26042815 PMC5885289

[63] Chander G, Monroe AK, Crane HM, HIV primary care providers: screening, knowledge, attitudes and behaviors related to alcohol interventions. Drug Alcohol Depend. 2016;161:59-66. doi:10.1016/j.drugalcdep.2016.01.015 26857898 PMC4841449

[64] Edelman EJ, Tetrault JM Unhealthy alcohol use in primary care: the elephant in the examination room. JAMA Intern Med. 2019;179(1):9-10. doi:10.1001/jamainternmed.2018.6125 30422276

[65] Bazzi A, Saitz R Screening for unhealthy alcohol use. JAMA. 2018;320(18):1869-1871. doi:10.1001/jama.2018.16069 30422181 PMC6296380

